# Severe Inflammatory Response and Moderate Acute Respiratory Distress Syndrome (ARDS) in an Immunocompetent Patient With Disseminated Tuberculosis: A Case Report

**DOI:** 10.7759/cureus.116708

**Published:** 2026-09-22

**Authors:** Rawan Zeizzoun, Linnet Leon Chaviano, Amer Zeizoun, Mahesh Kumar

**Affiliations:** 1 Faculty of Medicine, Ain Shams University, Cairo, EGY; 2 Internal Medicine, Broward Health North, Deerfeild Beach, USA; 3 Infectious Disease, Broward Health North, Deerfeild Beach, USA

**Keywords:** acute respiratory distress syndrome, adjunctive corticosteroids, disseminated tuberculosis, hyperinflammatory syndrome, immunocompetent host

## Abstract

An acute exacerbation in some tuberculosis (TB) cases can lead to widespread infection; this disseminated disease may progress when the body's immune response becomes dysregulated. Although rare, certain patients without obvious immune compromise can develop severe organ dysfunction if inflammation escalates uncontrollably. Respiratory failure can develop rapidly once lung tissue becomes overwhelmed by excessive inflammatory signaling. This presentation is uncommon but severe and is frequently unrecognized in its early stages because symptoms mimic other critical conditions. Mortality increases substantially once acute respiratory distress syndrome (ARDS) develops in the setting of disseminated infection.

A 40-year-old man, previously in good health, presented with severe respiratory distress. Oxygen saturation declined rapidly, requiring urgent endotracheal intubation. He subsequently required vasopressor support and prone positioning due to advanced respiratory failure. Laboratory evaluation revealed thrombocytopenia and elevated liver enzymes. Comprehensive microbiological testing did not identify common viral, bacterial, or fungal pathogens. A positive *Mycobacterium tuberculosis* (MTB) PCR result from bronchial fluid confirmed disseminated TB. His clinical course was complicated by moderate ARDS and a hyperinflammatory state, consistent with an exaggerated immune response to TB. Treatment was initiated with rifampin, isoniazid, pyrazinamide, and ethambutol (RIPE) therapy along with systemic corticosteroids (methylprednisolone 40 mg every eight hours). Clinical improvement was observed, and ventilatory support was discontinued following targeted treatment. The patient was discharged with a plan for prolonged anti-tubercular therapy. This case illustrates how disseminated TB can trigger a severe inflammatory response leading to moderate ARDS, even in the absence of underlying immunosuppression. Early recognition of this presentation is critical, and combined treatment with antimicrobial therapy and immunomodulation may improve outcomes.

## Introduction

Tuberculosis (TB) remains a major global health challenge, with substantial morbidity and mortality worldwide [1,2]. Recent global reports indicate TB diagnoses have risen to record levels, though mortality has shown some decline [2]. While the lungs are most often affected, the pathogen can occasionally spread beyond, triggering unusual and severe reactions elsewhere. Similar to viral infections such as influenza or SARS-CoV-2, overwhelming immune activation can occur, leading to excessive inflammatory signaling and tissue injury. This phenomenon, termed cytokine storm syndrome, was previously associated primarily with viral infections, but emerging evidence indicates it can also occur in bacterial infections such as TB [3,4]. Historically underappreciated in clinical practice, such exaggerated immune responses may contribute to rapid clinical deterioration. Delayed recognition of this presentation can delay treatment initiation and worsen outcomes. However, identifying this state at the bedside remains a challenge, as clinicians often lack an explicit diagnostic framework such as the hemophagocytic syndrome score (HScore) or hemophagocytic lymphohistiocytosis (HLH) 2004 criteria for TB-associated hyperinflammation [5,6]. The closely related, better-characterized entity of TB-associated HLH or macrophage activation syndrome (MAS) must be distinguished early to guide treatment.

The underlying pathophysiology involves dysregulated innate immune activation. Macrophages recognize *Mycobacterium tuberculosis* (MTB) through pattern recognition receptors, triggering downstream inflammatory cascades and the release of pro-inflammatory cytokines including TNF-α, IFN-γ, IL-1β, and IL-6 [4]. When this response becomes excessive and uncontrolled, it results in tissue injury, vascular leak, alveolar damage, and multiorgan dysfunction rather than effective pathogen containment. Clinically, this manifests as respiratory failure, hemodynamic instability, hepatic dysfunction, and hematologic abnormalities. Early recognition is critical, as the development of acute respiratory distress syndrome (ARDS) in this setting is associated with substantially increased mortality [7].

## Case presentation

A 40-year-old man presented to the emergency department with a three-week history of progressive dyspnea and persistent nonproductive cough that worsened in the recumbent position. He denied fever, chest pain, and chills. His epidemiologic history was notable for migration through Brazil and Mexico approximately three years prior to presentation, with repeated exposure to contaminated water sources. He reported no history of alcohol consumption, tobacco smoking, or intravenous drug use. He had been incarcerated in Haiti approximately four to five years earlier. The patient’s childhood Bacille Calmette-Guérin (BCG) vaccination status was unknown, providing relevant clinical context for the initial negative screening assays.

On two prior occasions, he had been admitted for management of a serious wound on his right foot. During those stays, tissue samples grew* Proteus, Enterobacter,* and *Arcanobacterium*. During the second admission, computed tomography (CT) of the chest identified an 8 mm ground-glass opacity in the right upper lobe; imaging from prior hospitalizations was not available for inclusion in this report. He was discharged with a four-week course of amoxicillin-clavulanate and trimethoprim-sulfamethoxazole, which he discontinued prematurely.

Initial presentation and hospital course (days 1-2)

On presentation, the patient was in acute distress with hypoxemic respiratory failure. He was tachycardic (heart rate (HR) 110 beats per minute), hypotensive (blood pressure (BP) 97/55 mmHg), and hypoxic. He was initially placed on bilevel positive airway pressure (BiPAP) but rapidly deteriorated, requiring emergent endotracheal intubation, mechanical ventilation, and initiation of norepinephrine for vasopressor support. He was sedated, paralyzed, and placed in the prone position for moderate ARDS management (Figure 1). At the time of the reported blood gas, the patient was ventilated on a volume-controlled mode with a tidal volume of 6 mL/kg, a fraction of inspired oxygen (FiO_2_) of 1.0, and a positive end-expiratory pressure (PEEP) of 10 cmH_2_O.

**Figure 1:**
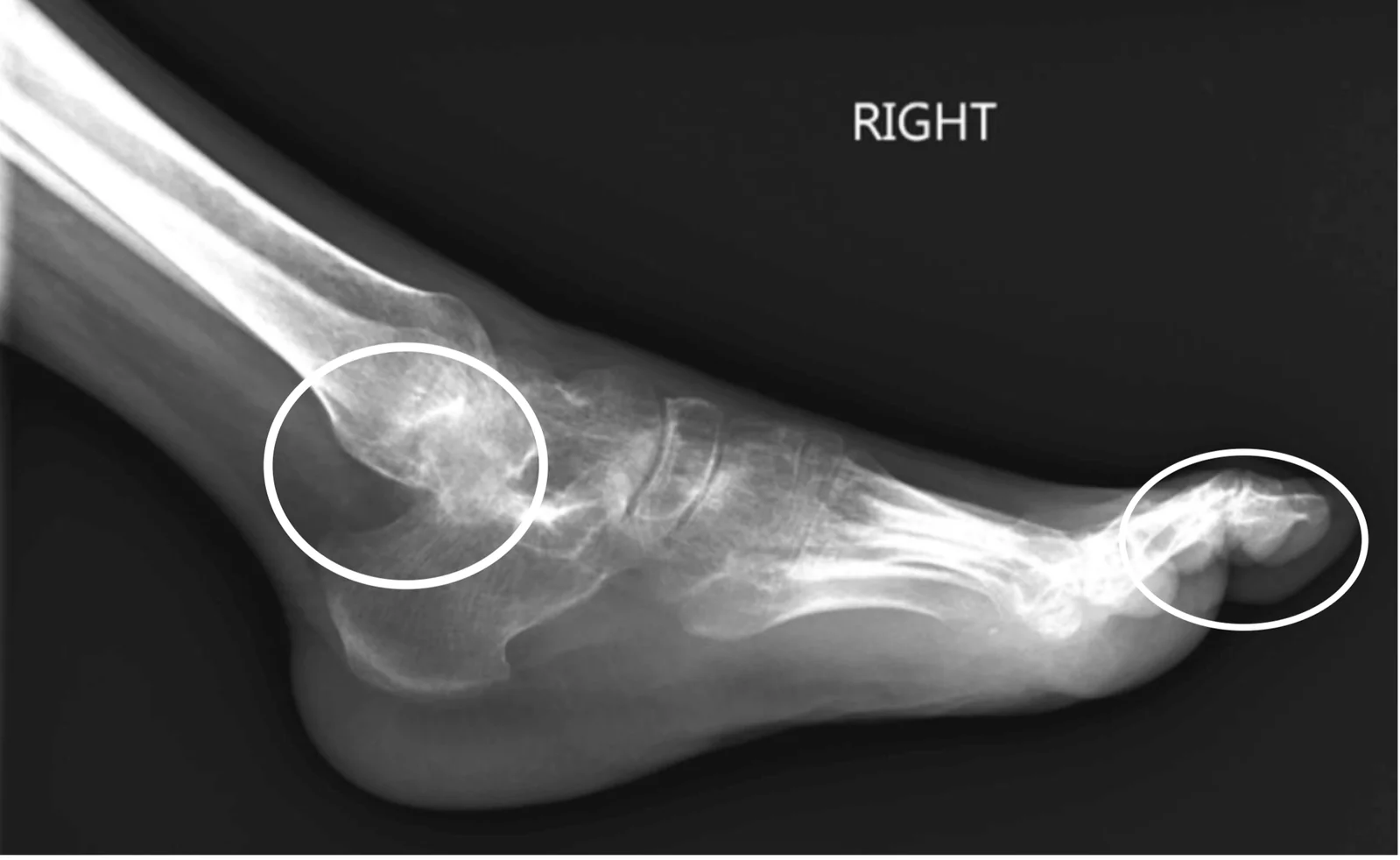
Lateral radiograph of the right foot demonstrating chronic osseous deformity and associated soft-tissue abnormalities. The left circle highlights soft-tissue thickening involving the midfoot region, while the right circle depicts chronic deformity of the forefoot with bony irregularity.

Physical Examination

The patient was intubated and sedated. The right foot showed a large plantar medial wound (15 × 5 cm) with well-demarcated borders, beefy red granulation tissue, and minimal surrounding inflammation (Figure 2).

**Figure 2:**
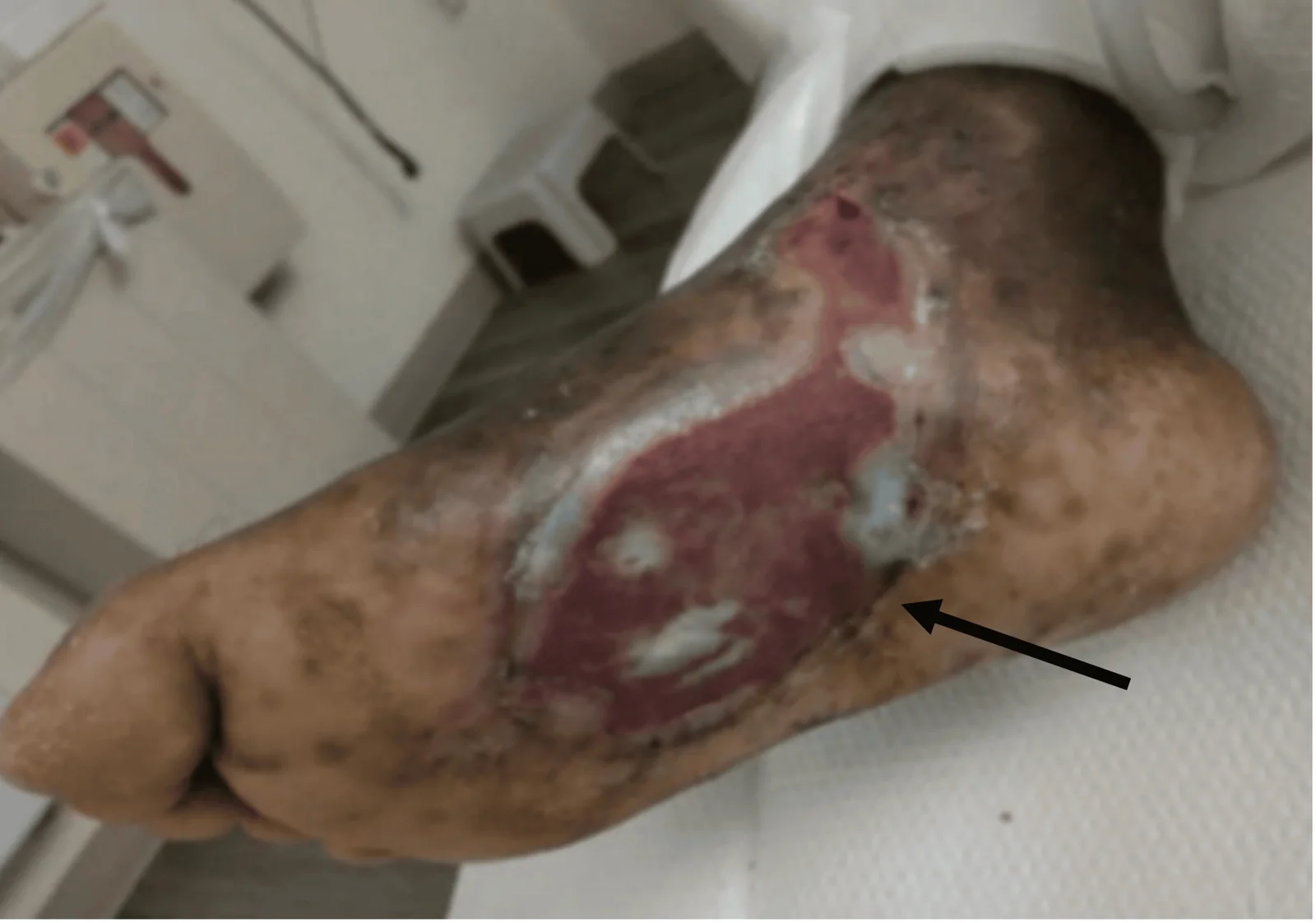
Plantar medial wound on the right foot measuring approximately 15 × 5 cm, extending to the medial malleolus. The wound has well-demarcated borders and extends down to the subcutaneous tissue. The wound bed shows beefy red granulation tissue with small areas of white slough. There is no surrounding erythema, edema, or warmth. The arrow highlights the area of superficial slough within the wound bed.

Laboratory Assessment

Initial laboratory evaluation demonstrated severe thrombocytopenia (platelets 31 ×10^3^/µL) and microcytic anemia (hemoglobin 12.8 g/dL, mean corpuscular volume (MCV) 59.4 fL), along with transaminitis (aspartate aminotransferase (AST) 256 U/L, alanine aminotransferase (ALT) 194 U/L), hypoalbuminemia (albumin 3.0 g/dL), and metabolic acidosis (bicarbonate 18 mmol/L), as detailed in Table 1.

**Table 1:** Laboratory findings on admission WBC: White blood cell count; MCV: Mean corpuscular volume; MCH: Mean corpuscular hemoglobin; MCHC: Mean corpuscular hemoglobin concentration; RDW: Red cell distribution width; BUN: Blood urea nitrogen; AST: Aspartate aminotransferase; ALT: Alanine aminotransferase; eGFR: Estimated glomerular filtration rate; LDH: Lactate dehydrogenase; PT INR: Prothrombin time international normalized ratio; APTT: Activated partial thromboplastin time; PaO_2_/FiO_2_: Ratio of partial pressure of arterial oxygen to fraction of inspired oxygen; pO_2_: Partial pressure of oxygen; ABG: Arterial blood gas; ANA: Antinuclear antibody; RPR: Rapid plasma reagin; AFB: Acid-fast bacilli; Ag: Antigen; BAL: Bronchoalveolar lavage; MTB: *Mycobacterium tuberculosis*; TB: Tuberculosis

Test	Patient Value	Normal Range	Interpretation
Hematology			
WBC	1.82 × 10³/µL	4.5-11.0 × 10³/µL	Severe leukopenia
Hemoglobin	12.8 g/dL	13.5-17.5 g/dL	Mild anemia
Hematocrit	32.40%	41-53%	Low
MCV	59.4 fL	80-100 fL	Microcytic
MCH	17.4 pg	27-33 pg	Low
MCHC	29.3 g/dL	32-36 g/dL	Low
RDW	23.70%	11.5-14.5%	Elevated
Platelets	31 × 10³/µL	150-400 × 10³/µL	Severe thrombocytopenia
Metabolic Panel			
Sodium	133 mEq/L	136-145 mEq/L	Mild hyponatremia
Potassium	3.5 mEq/L	3.5-5.0 mEq/L	Low normal
Bicarbonate	18 mmol/L	22-29 mmol/L	Metabolic acidosis
BUN	13 mg/dL	7-25 mg/dL	Normal
Creatinine	0.7 mg/dL	0.7-1.3 mg/dL	Normal
Glucose	103 mg/dL	70-99 mg/dL	Mildly elevated
Calcium	8.4 mg/dL	8.5-10.2 mg/dL	Low normal
Anion Gap	7 mEq/L	8-16 mEq/L	Normal
Liver Function			
AST	256 U/L	10-40 U/L	Markedly elevated
ALT	194 U/L	7-56 U/L	Elevated
Alkaline Phosphatase	95 U/L	44-147 U/L	Normal
Total Bilirubin	0.7 mg/dL	0.2-1.2 mg/dL	Normal
Total Protein	6.2 g/dL	6.3-8.2 g/dL	Low normal
Albumin	3.0 g/dL	3.5-5.0 g/dL	Hypoalbuminemia
eGFR	117 mL/min	>60 mL/min	Normal
Inflammatory Markers			
LDH	512 U/L	140-280 U/L	Elevated
Ferritin	112 ng/mL	24-336 ng/mL	Normal
Procalcitonin	0.12 ng/mL	<0.25 ng/mL	Normal
Coagulation			
PT INR	1.1	0.8-1.2	Normal
APTT	33.2 seconds	25-35 seconds	Normal
Fibrinogen	312 mg/dL	200-400 mg/dL	Normal
Arterial Blood Gas (Post-intubation)			
pH Arterial	7.33	7.35-7.45	Mild acidemia
pO_2_ Arterial	139 mmHg	80-100 mmHg	On FiO_2_ 100% ventilator
PaO_2_/FiO_2_ ratio	139 mmHg	>300 mmHg	Moderate ARDS - Berlin criteria
Bicarbonate (ABG)	22.2 mmol/L	22-26 mmol/L	Normal
Additional Workup			
HIV	Non-reactive	-	Negative
Hepatitis A, B, C	Non-reactive	-	Negative
ANA Screen	Negative	-	Negative
RPR	Non-reactive	-	Negative
Histoplasma Urine Ag	Negative	-	Negative
Legionella Urine Ag	Negative	-	Negative
Blood Cultures	No growth	-	Negative
Fungal Cultures	Negative	-	Negative
QuantiFERON-TB Gold	Negative	-	Negative
AFB Smear	Negative	-	Negative
MTB-PCR (BAL)	Detected	-	Confirmed TB diagnosis
Sputum AFB Culture	Positive - MTB complex	-	Confirmed TB diagnosis

Infectious Workup (Comprehensive)

All results were negative, including multiplex PCR panels for respiratory viruses (influenza, respiratory syncytial virus (RSV), SARS-CoV-2, etc.), bacterial pathogens (*Mycoplasma*, *Streptococcus pneumoniae *antigen), fungal cultures, and blood cultures. QuantiFERON-TB Gold (QIAGEN, Germantown, USA) was negative [8]. Acid-fast bacilli (AFB) stain on concentrated smear was negative [9].

Imaging

Chest X-ray showed diffuse bilateral pulmonary opacities consistent with ARDS (Figure 3). CT chest demonstrated extensive bilateral ground-glass opacities, multifocal consolidations, cavitary lesions, and predominant upper-lobe involvement (Figures 4-6).

**Figure 3:**
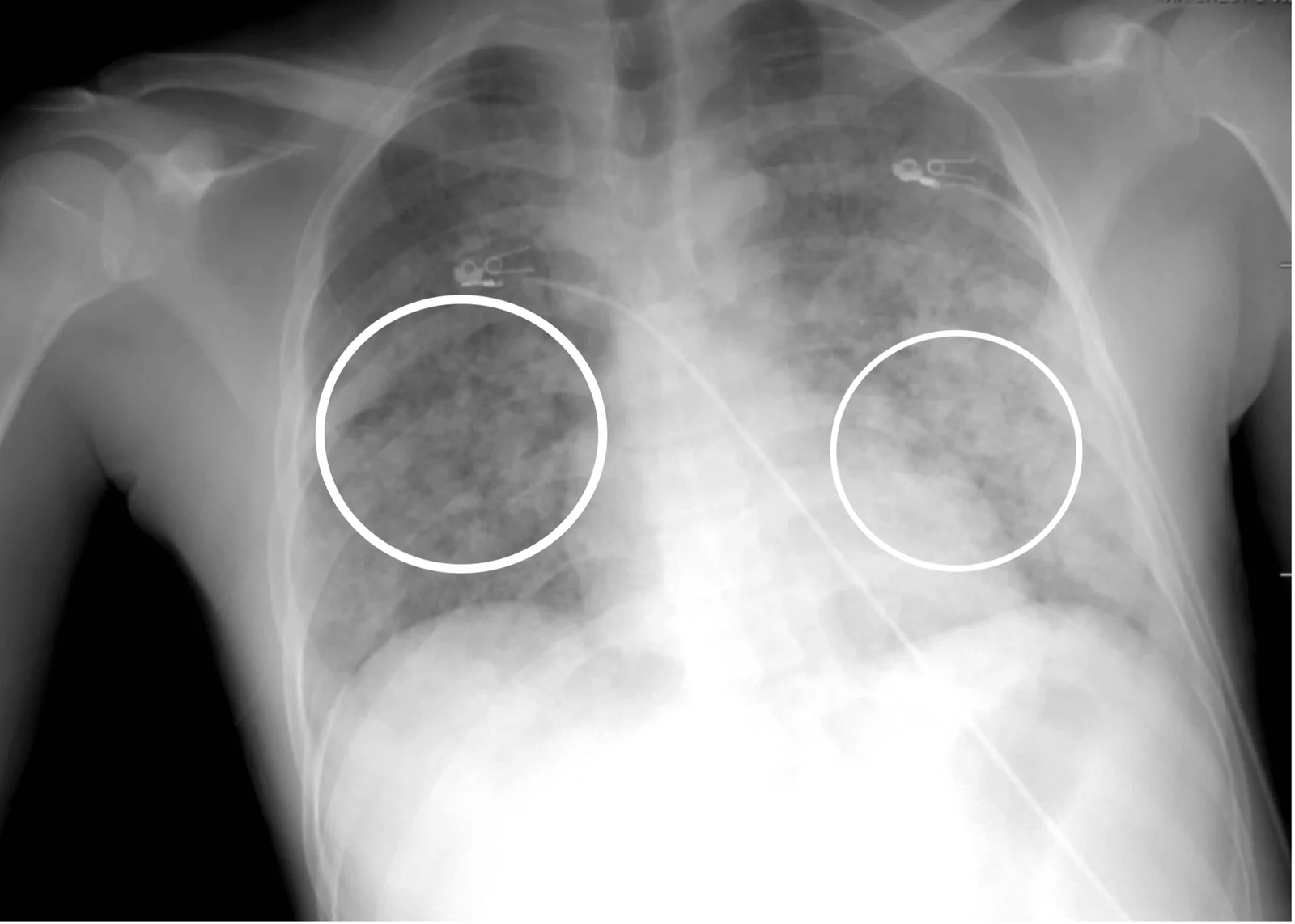
Admission chest radiograph demonstrating extensive bilateral airspace opacities and consolidation consistent with severe diffuse pulmonary involvement. The circles highlight widespread bilateral parenchymal opacities involving the mid-to-lower lung zones, corresponding to areas of significant inflammatory consolidation.

**Figure 4:**
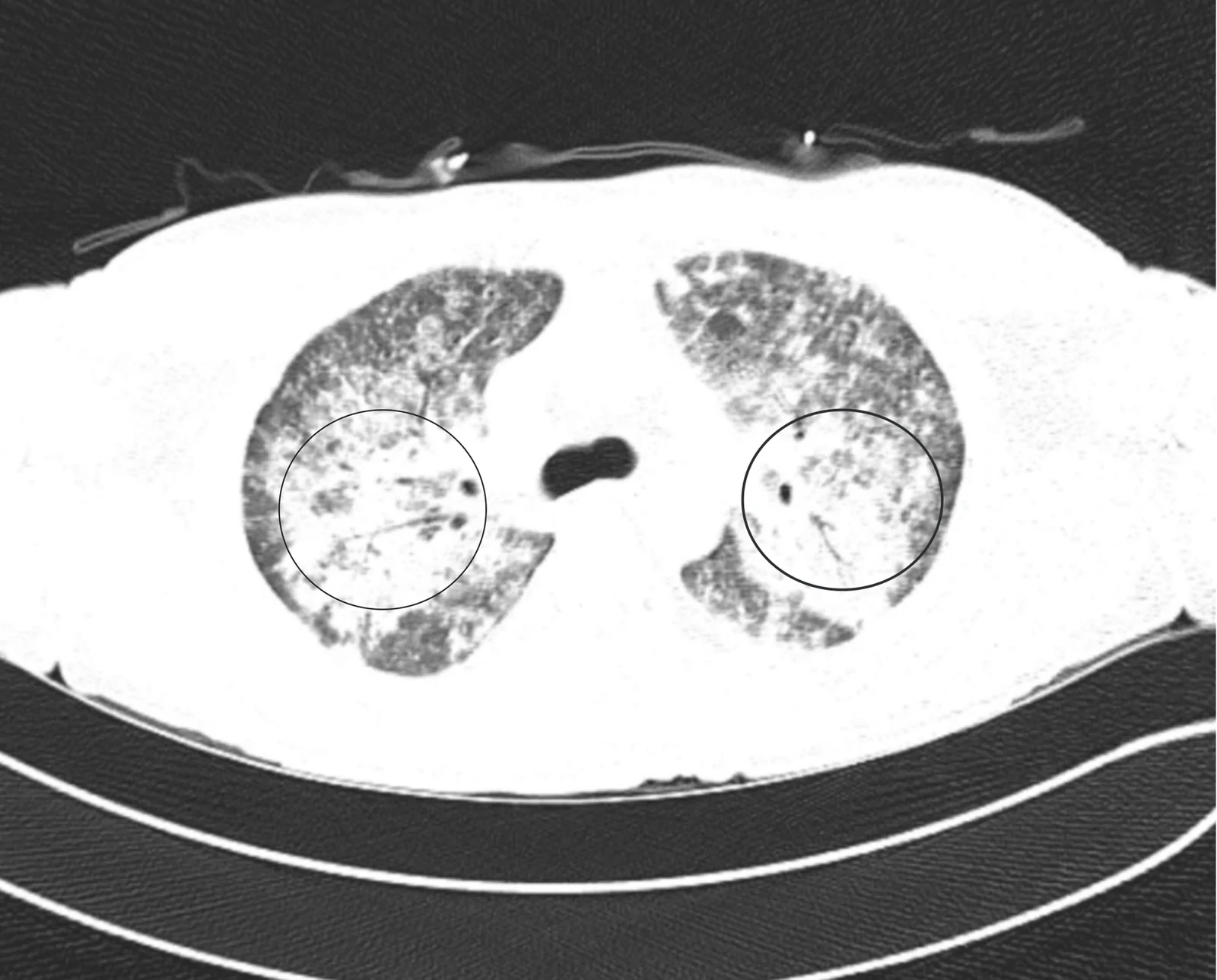
Axial CT chest image demonstrating diffuse bilateral ground-glass opacities and confluent consolidations consistent with advanced pulmonary disease. The circles delineate bilateral cavitary foci embedded within areas of dense inflammatory consolidation. CT: Computed tomography

**Figure 5:**
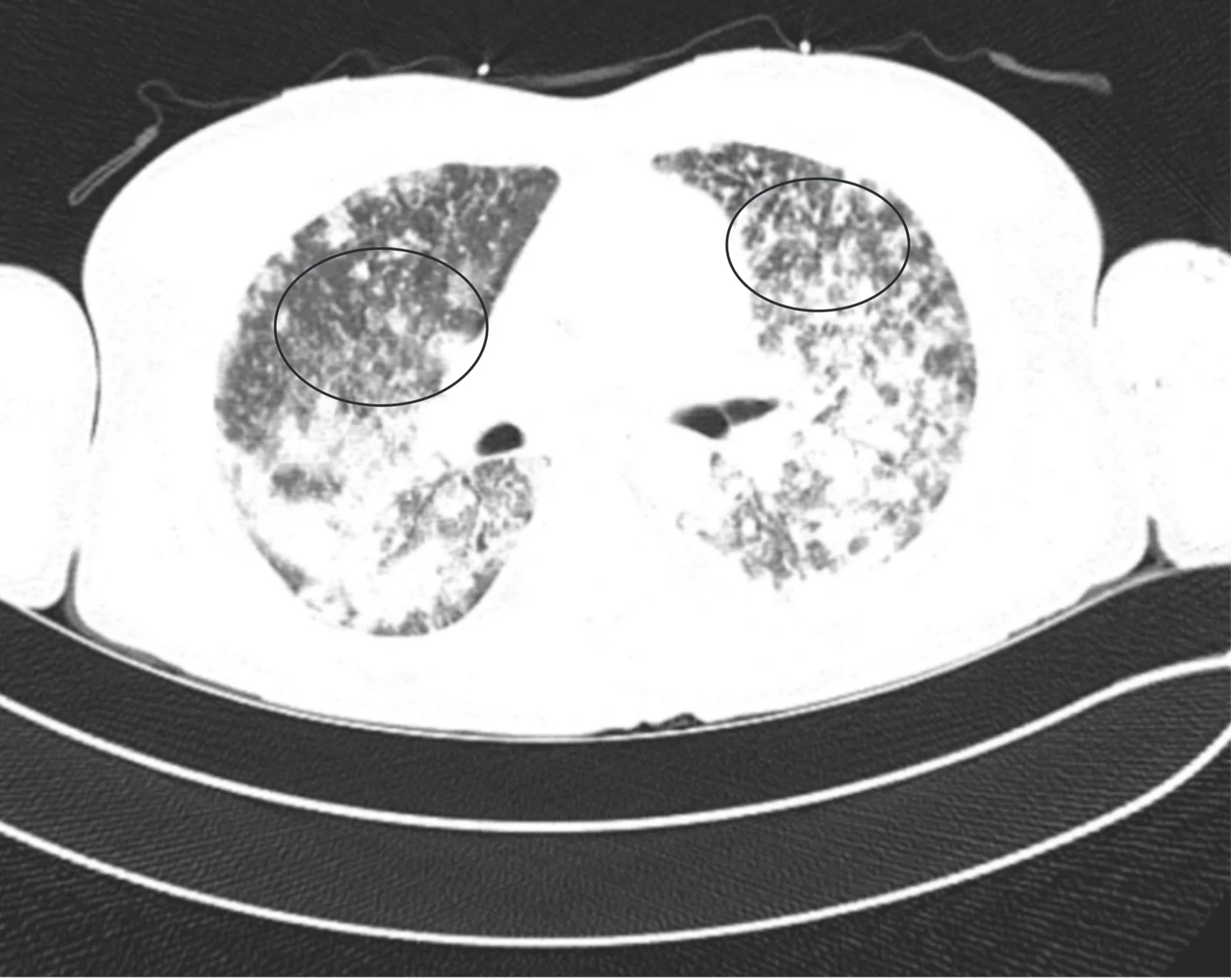
Coronal CT image demonstrating diffuse, predominantly upper-lobe pulmonary involvement consistent with active tuberculosis. The circles delineate bilateral upper-lobe parenchymal infiltrates characterized by confluent ground-glass opacities and areas of consolidation consistent with active tuberculosis. CT: Computed tomography

**Figure 6:**
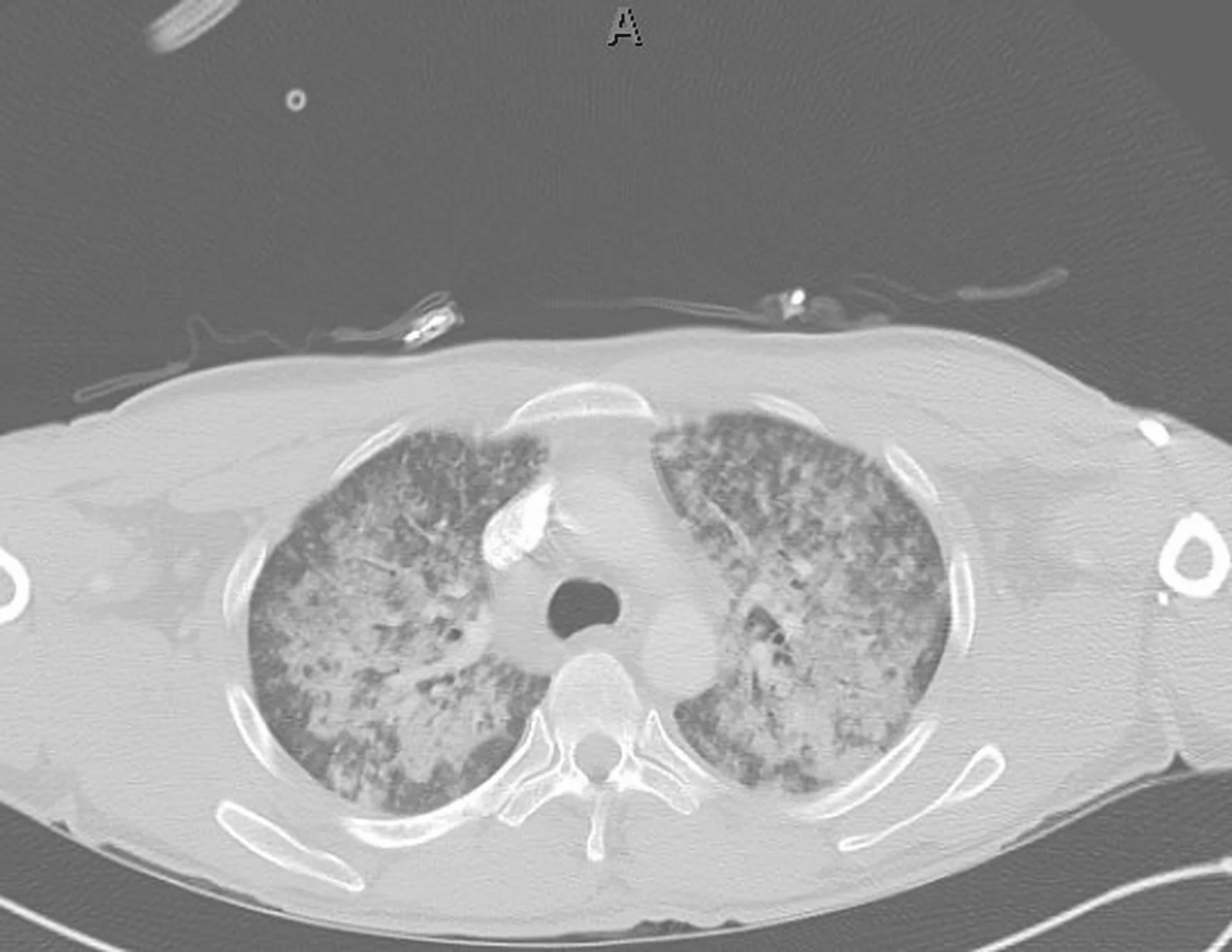
Axial CT chest image demonstrating diffuse bilateral ground-glass opacities and confluent consolidations with scattered nodular densities, consistent with extensive pulmonary tuberculosis. CT: Computed tomography

Given the severity of his presentation and unknown etiology, empiric therapy was started on hospital day 2 with meropenem, vancomycin, Bactrim (possible *Pneumocystis jirovecii* pneumonia (PJP)), and amphotericin B. Bronchoscopy with BAL was performed.

Diagnosis and targeted therapy (day 3)

On hospital day 3, MTB-PCR from BAL returned positive for MTB complex; all other BAL microbial studies remained negative.

A diagnosis of disseminated TB with associated severe inflammatory response and moderate ARDS was made. Antituberculous therapy was initiated immediately according to international standards for TB care [10], consisting of rifampin 600 mg intravenously daily, isoniazid 300 mg via nasogastric tube daily, pyrazinamide 1500 mg via nasogastric tube daily, and ethambutol 1600 mg via nasogastric tube daily. Levofloxacin 750 mg intravenously daily was added because central nervous system involvement could not be initially excluded. Adjunctive immunomodulatory therapy with methylprednisolone 40 mg intravenously every eight hours was initiated concurrently given the severity of the hyperinflammatory response [9].

Clinical response and further workup

Following initiation of antituberculous therapy and corticosteroids, the patient demonstrated progressive clinical improvement. Vasopressor requirements resolved, oxygenation improved, and mechanical ventilation was successfully discontinued, with the patient transitioned to supplemental oxygen via nasal cannula.

To evaluate for extrapulmonary dissemination, magnetic resonance imaging (MRI) of the brain was performed and was unremarkable, effectively excluding central nervous system involvement (Figure 7). Bone marrow biopsy demonstrated subtle non-necrotizing granulomata within a normocellular marrow with intact trilineage hematopoiesis, confirming hematogenous dissemination of TB (Figure 8). Histopathological slide images were not available for inclusion in this report. Importantly, a retrospective review of the core pathology specimen confirmed that hemophagocytosis was explicitly absent.

**Figure 7:**
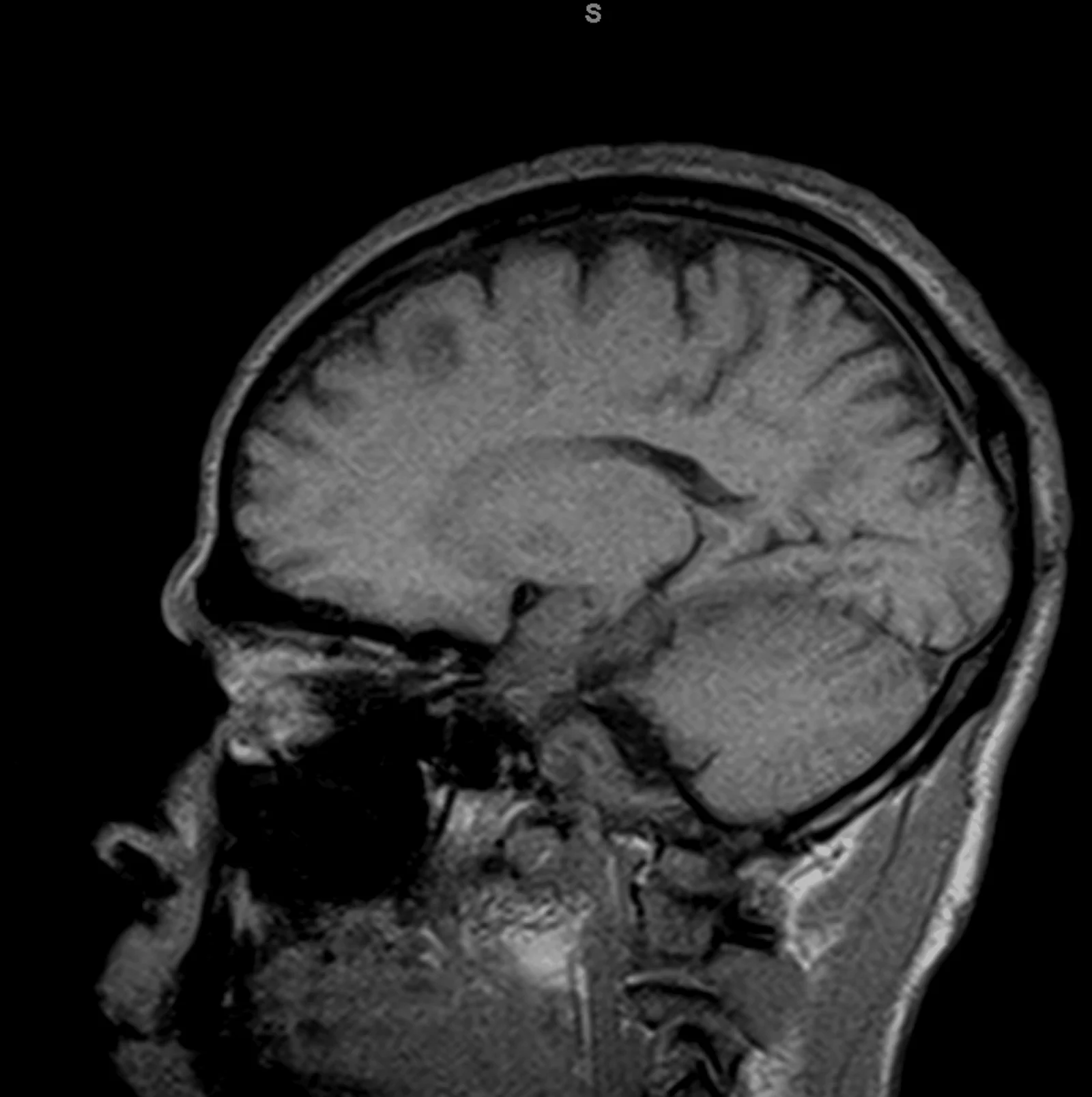
Sagittal T1-weighted MRI of the brain demonstrating no focal lesions, abnormal signal, or meningeal enhancement. Findings are within normal limits, excluding central nervous system dissemination. MRI: Magnetic resonance imaging

**Figure 8:**
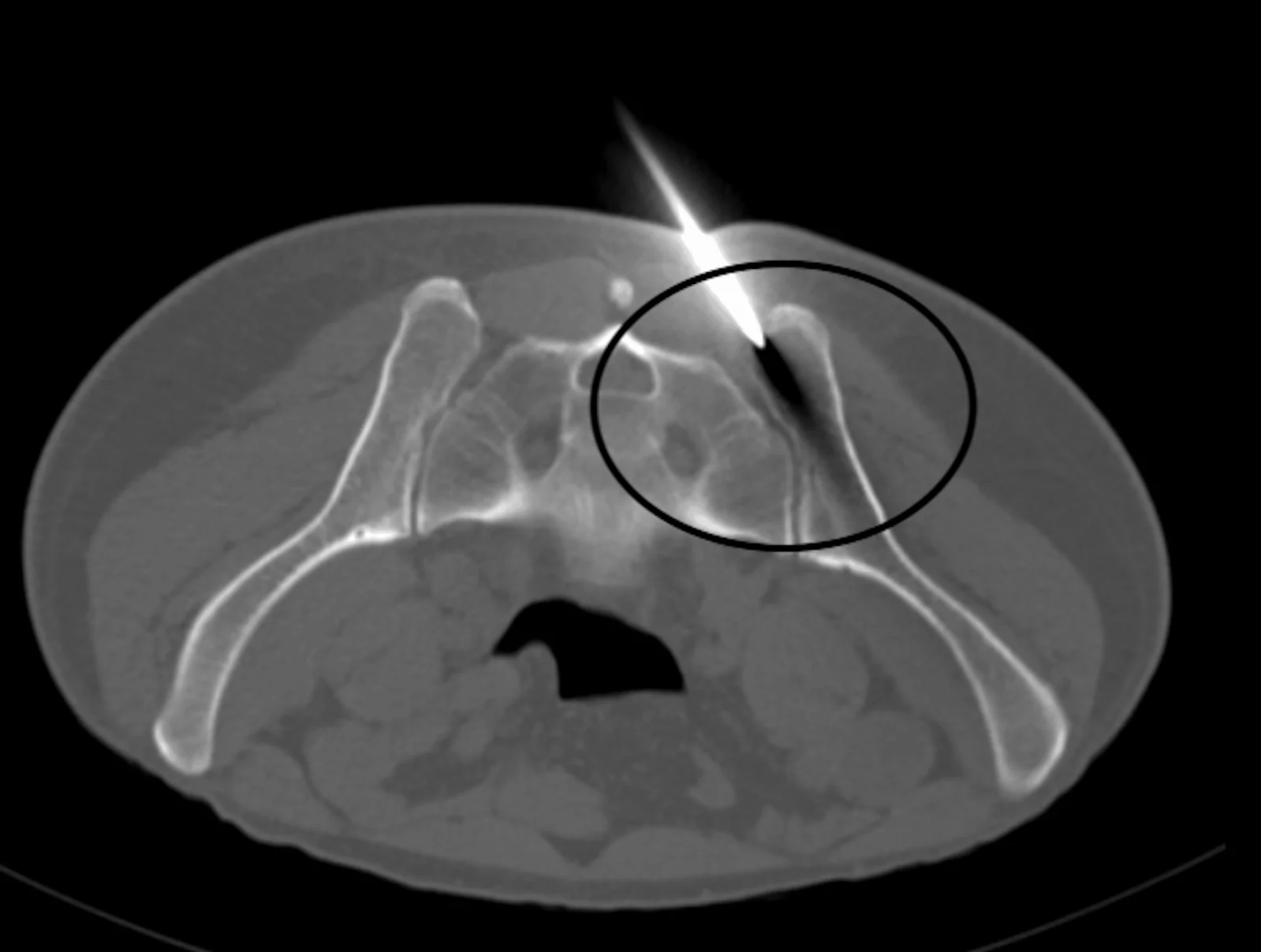
CT-guided posterior iliac bone marrow biopsy demonstrating needle placement for tissue sampling. The circle depicts the needle tip. Pathological examination of the core biopsy specimen revealed subtle non-necrotizing granulomata within normocellular marrow with intact trilineage hematopoiesis, consistent with hematogenous dissemination of tuberculosis. Microscopic slide images were not available for inclusion in this report. CT: Computed tomography

Empiric antimicrobial therapy was administered for 14 days, overlapping with the initiation of antituberculous therapy, before being discontinued upon confirmed microbiologic diagnosis. The patient was hospitalized for a total of 15 days before being discharged in stable condition.

Outcome

His clinical improvement was sustained. Inflammatory markers fell; thrombocytopenia and transaminitis improved, as reflected in Table 2.

**Table 2:** Laboratory trends during hospitalization demonstrating clinical improvement following initiation of antituberculous therapy and corticosteroids WBC: White blood cell count; AST: Aspartate aminotransferase; ALT: Alanine aminotransferase; PaO_2_/FiO_2_: Ratio of partial pressure of arterial oxygen to fraction of inspired oxygen

Test	Admission	During Hospitalization	At Discharge	Normal Range	Trend
WBC	1.82 × 10³/µL	2.78 × 10³/µL	-	4.5-11.0 × 10³/µL	Improving
Neutrophils	-	54%	-	50-70%	Normal
Lymphocytes	-	34%	-	20-40%	Normal
Hemoglobin	12.8 g/dL	9.7 g/dL	-	13.5-17.5 g/dL	Stable
Hematocrit	32.40%	33.80%	-	41-53%	Stable
Platelets	31 × 10³/µL	115 × 10³/µL	-	150-400 × 10³/µL	Significant improvement
AST	256 U/L	50 U/L	33 U/L	10-40 U/L	Significant improvement
ALT	194 U/L	81 U/L	53 U/L	7-56 U/L	Significant improvement
Albumin	3.0 g/dL	3.4 g/dL	3.5 g/dL	3.5-5.0 g/dL	Normalized
Sodium	133 mEq/L	135 mEq/L	-	136-145 mEq/L	Improving
Bicarbonate	18 mmol/L	24 mmol/L	-	22-29 mmol/L	Normalized
Glucose	103 mg/dL	115 mg/dL	160 mg/dL	70-99 mg/dL	Elevated
PaO_2_/FiO_2_	139 mmHg	267 mmHg	-	>300 mmHg	Significant improvement
pH Arterial	7.33	7.27	-	7.35-7.45	Stabilizing

At discharge, therapy transitioned to oral TB treatment and a gradually reducing prednisone taper, coordinated with infectious disease specialists and local health authorities.

## Discussion

The immune response to MTB represents a balance between protection and pathology. A strong type 1 T helper (Th1) response supports granuloma formation and limits dissemination, but when dysregulated, it can cause significant tissue injury through excessive inflammation. During disseminated infection, high antigenic burden can overstimulate macrophages and T cells, resulting in excessive cytokine production. High concentrations of IL-6 and other mediators have been linked to worse outcomes in TB. These inflammatory elements may contribute to vascular leak, alveolar injury resembling ARDS, hepatic dysfunction, and hematologic abnormalities.

Diagnostic challenges and hyperinflammatory frameworks

The diagnosis was challenging for several reasons. First, the presence of an apparently intact immune system may lower clinical suspicion for a hyperinflammatory response to infection. Second, IFN-γ release assays and AFB smears can be negative in active disease, particularly in severe or disseminated presentations. In this setting, the decisive step was molecular testing via MTB-PCR, which is capable of detecting TB even when smear results remain negative.

Radiologic findings added further ambiguity. The combination of tree-in-bud nodularity and a cavitary nodule initially raised concern for atypical bacterial pneumonia or fungal infection, rather than TB. This underscores how imaging alone can mislead clinical suspicion in the absence of molecular confirmation.

Crucially, while our patient presented with hallmarks of an intense systemic response - including severe leukopenia, thrombocytopenia, and transaminitis - standard laboratory markers for classic cytokine storm syndromes or HLH/MAS were not fully realized. His ferritin (112 ng/mL) and fibrinogen (312 mg/dL) remained strictly within normal reference intervals, and validated diagnostic scoring frameworks such as the HScore or HLH-2004 criteria could not be met [5,6]. In settings of disseminated TB, HLH/MAS represents the recognized nosological entity driving hematologic collapse. Our patient’s bone marrow biopsy, which demonstrated subtle non-necrotizing granulomata despite normal ferritin, indicates that profound hematogenous dissemination can prompt severe organ injury and ARDS without manifesting the complete, classic laboratory phenotype of systemic hyperinflammation.

ARDS classification and the role of corticosteroids

Based on the objective PaO_2_/FiO_2_ ratio of 139 mmHg obtained while the patient was on mechanical ventilation with a PEEP of 10 cmH_2_O, the respiratory failure satisfies the Berlin definition for moderate ARDS [11]. This case is also noteworthy when evaluated under the 2023 global definition of ARDS [12], which expands diagnostic parameters to include patients managed with high-flow nasal oxygen (HFNO) ≥30 L/min or non-invasive ventilation in resource-limited settings, tracking closely with our patient’s step-down recovery phase.

Management of suspected TB-associated hyperinflammation requires both effective antimicrobial therapy and suppression of the excessive inflammatory response. High-dose intravenous methylprednisolone was initiated alongside antitubercular therapy in this case, aligning with evidence supporting steroid utility in selected severe TB manifestations [13]. In selected severe pulmonary inflammatory conditions, corticosteroids have been associated with improved outcomes [14]. The relatively rapid clinical improvement following the initiation of corticosteroids demonstrates a strong temporal association that suggests immune dysregulation, rather than infection alone, contributed substantially to the patient's acute deterioration [15,16].

Alternative portals of entry

The presence of a chronic foot wound with potential environmental exposure is noteworthy. TB typically enters through inhalation, but disrupted skin barriers in high-risk environments theoretically raise the possibility of atypical entry or an unrecognized early focus, with later dissemination. Despite prior cultures yielding common bacterial pathogens, alternative hypotheses remain plausible; slow-growing mycobacteria may have seeded the extremity wound bed earlier or spread hematogenously at a later stage [17]. The preexisting right upper lobe ground-glass opacity could also represent an earlier primary pulmonary focus that subsequently progressed subclinically. Organ dysfunction remains a major determinant of mortality in critical illness, especially when inflammatory cascades become dysregulated [18].

## Conclusions

This case highlights that disseminated TB should be considered in patients presenting with moderate ARDS and secondary hyperinflammatory features, even in the absence of classical immunosuppression or early positive TB screening tests. While direct, serial cytokine measurements were unavailable to definitively quantify the hyperinflammatory state, the clinical phenotype was highly characteristic. When respiratory failure, thrombocytopenia, and hepatic dysfunction occur together, particularly in patients from TB-endemic regions, an overwhelming immune response to mycobacterial infection should be included in the differential diagnosis. Prompt initiation of multidrug antituberculous therapy combined with adjunctive corticosteroids may improve outcomes, as suggested by this case.
